# Hospital Indicators for the Management of Native and Prosthetic Valve Infective Endocarditis Across Brazilian Regions From 2015 to 2024

**DOI:** 10.7759/cureus.117169

**Published:** 2026-09-29

**Authors:** Lívia L Marques, Lívia Kobarg c Akerman, Luiza M Diniz, Louise M da Rocha Sady, Selena M Brandão, Tuany C Bernardi, Lucas F Ananias

**Affiliations:** 1 Medicine, Pontifícia Universidade Católica de Minas Gerais, Contagem, BRA; 2 Medicine, Universidade Alto Vale do Rio do Peixe, Caçador, BRA; 3 Medicine, Universidade Federal De São João Del Rei, Divinópolis, BRA; 4 General Medicine, Centro Universitário Facid Wyden, Teresina, BRA; 5 Medicine, Universidade de Uberaba, Uberaba, BRA; 6 Medicine, Universidade Paranaense, Umuarama, BRA; 7 Tropical Medicine and Infectious Diseases, Universidade Federal do Triângulo Mineiro, Uberaba, BRA

**Keywords:** ecological studies, health information systems, heart valve prosthesis, hospital mortality, infective endocarditis

## Abstract

Objective: This study aimed to analyze regional distributions and temporal trends in approved Hospital Admission Authorizations (AIHs), in-hospital deaths, proportions of admissions resulting in in-hospital death, and length of hospital stay associated with hospital care for native-valve and prosthetic-valve infective endocarditis in Brazil from 2015 to 2024.

Methods: This retrospective ecological time-series study used aggregated data from the Brazilian Unified Health System Hospital Information System, accessed through DATASUS, for 2015-2024. Approved AIHs linked to selected procedure codes for native-valve and prosthetic-valve infective endocarditis treatment were analyzed across Brazil’s five regions, classified by hospital location. Descriptive measures included frequencies, deaths per 100 approved AIHs, mean hospital stay, and dispersion. Annual AIH and recorded in-hospital death rates per 100,000 population were calculated using year-specific regional population estimates. Trends in these rates were assessed using the Joinpoint Regression Program (version 6.0.1; U.S. National Cancer Institute, Bethesda, MD), estimating annual percentage changes (APCs) and average APCs with 95% confidence intervals from 10 annual observations per series. Statistical significance was set at α = 0.05.

Results: Native-valve treatment records comprised 13,920 approved AIHs and 1,966 in-hospital deaths (14.1 deaths per 100 AIHs), with authorizations concentrated in the Southeast and Northeast. AIH rates per 100,000 population declined significantly in the Northeast during 2015-2019 and increased in the Southeast during 2020-2024. Recorded in-hospital death rates per 100,000 population increased significantly in the Southeast and South. Prosthetic-valve treatment records comprised 6,610 approved AIHs and 1,115 in-hospital deaths (16.9 deaths per 100 AIHs). AIH rates fluctuated in the North and increased significantly in the Southeast during 2021-2024. No statistically significant regional trends were identified in recorded in-hospital death rates for this treatment category.

Conclusion: Hospital records linked to the selected infective endocarditis treatment procedure codes showed heterogeneous regional trends and a higher cumulative number of in-hospital deaths per 100 approved AIHs for prosthetic-valve treatment. These findings characterize recorded hospital utilization within SUS and may inform regional service monitoring and planning.

## Introduction

Infective endocarditis (IE) is an infection of the endocardial surface, most commonly involving native or prosthetic heart valves, and remains one of the most severe cardiovascular infections, with high morbidity and mortality rates [1,2]. Over recent decades, its global incidence has increased markedly, reaching an age-standardized rate of 13.8 cases per 100,000 population in 2019, with particularly high rates reported in parts of Latin America and substantial changes in the epidemiological profile of the disease [1,3].

IE now predominantly affects older adults, who frequently have degenerative valvular disease and a history of invasive cardiac procedures. In this context, prosthetic valve endocarditis (PVE) accounts for approximately 25%-30% of cases, reflecting the increasing number of valvular interventions and population aging [1,4,5]. Despite advances in diagnostic and therapeutic approaches, including more sensitive imaging modalities and improved surgical techniques, in-hospital mortality remains high, particularly in low- and middle-income countries [2,4]. Compared with native valve endocarditis (NVE), PVE poses greater diagnostic challenges and is associated with poorer clinical outcomes, including higher in-hospital mortality rates [5,6].

In Brazil, although most cases still involve native valves, the number of hospital admissions recorded for prosthetic-valve IE has increased over time. However, national evidence remains largely derived from tertiary referral centers, limiting the understanding of regional differences in disease occurrence and clinical outcomes [2,3].

Against this background, this study aimed to analyze the regional distribution and temporal trends in hospital admissions and in-hospital deaths associated with the treatment of IE in Brazil between 2015 and 2024, separately examining native-valve and prosthetic-valve cases. Data were obtained from the Brazilian Unified Health System Hospital Information System (SIH/SUS) and accessed through the TabNet/DATASUS platform.

## Materials and methods

A national ecological time series study that examined hospital care associated with native-valve and prosthetic-valve IE in Brazil from 2015 to 2024. The analytical unit was the region-year-procedure combination, comprising 100 aggregate observations across five geographic regions, 10 years, and two procedure categories. The regions were North, Northeast, Southeast, South, and Central-West. Each regional procedure-specific time series comprised 10 annual observations.

Hospital data were obtained from the SIH/SUS, accessed through TabNet/DATASUS in March 2026. The source was the hospital procedures database organized by hospital location. Tabulations were extracted by geographic region, processing year, and procedure, using the contents “approved Hospital Admission Authorizations” (AIHs), “deaths,” “inpatient days,” and “mean length of stay.” Geographic classification therefore reflected the location of the treating hospital. Processing year represented the administrative reporting period and did not necessarily correspond to the year of admission or disease onset [7].

Hospital care was identified using two codes from the Brazilian Unified Health System Table of Procedures, Medications, Orthoses, Prostheses, and Special Materials (SIGTAP): treatment of IE involving a native valve (03.03.06.016-6) and treatment of IE involving a prosthetic valve (03.03.06.015-8). All approved AIHs and associated outcomes recorded under these procedures during 2015-2024 were included. Records associated with other procedures or periods were outside the study scope, and rejected AIHs were absent from the approved totals. Case identification relied on administrative procedure codes, without independent clinical adjudication. Consequently, the study did not capture IE hospitalizations recorded exclusively under other codes.

Approved AIHs are administrative authorizations and may include both new admissions and extensions of prolonged hospital stays. Readmissions and transfers that generated additional approved authorizations remained included in the source totals. Because only aggregate tabulations were available, patient-level and authorization-level identifiers could not be used to identify first admissions, link readmissions, consolidate transfers, or remove potential duplicate authorizations. AIH counts therefore did not represent unique patients or independently identified episodes of IE.

The aggregate data were organized in a spreadsheet containing year, geographic region, procedure category, approved AIHs, in-hospital deaths, and source-reported mean length of stay. Structural checks assessed completeness and uniqueness of the region-year-procedure combinations. All 100 combinations were present, without duplicate aggregate rows or missing values in these variables; no imputation was required. These checks could not determine whether repeated authorizations or hospitalizations occurred within the underlying administrative records.

Population denominators were obtained from the resident population estimates disseminated through TabNet/DATASUS in the table “População residente - estudo de estimativas populacionais por município, idade e sexo.” These estimates are produced by the Brazilian Ministry of Health using demographic data from the Brazilian Institute of Geography and Statistics. The population of each region and year from 2015 to 2024 was used as the denominator. The source defines resident population as the estimated population on July 1 of the reference year [8].

Annual approved-AIH rates and in-hospital death rates were defined as the respective counts divided by the resident population of the corresponding region and year, multiplied by 100,000. Because hospital events were classified by hospital location, the rates described regional hospital activity relative to the resident population and could be influenced by interregional referral. They did not measure the incidence of IE among residents. Deaths represented in-hospital deaths associated with the selected procedure records; IE was not independently established as the underlying cause of death.

In-hospital deaths per 100 approved AIHs were calculated by dividing the number of deaths by the number of approved AIHs within the same region, year, and procedure category and multiplying by 100. Cumulative percentages for 2015-2024 used the ratio of summed deaths to summed approved AIHs. We calculated national percentages after aggregating regional counts. These measures used absolute counts rather than ratios of rounded population-based rates or arithmetic means of annual percentages. Because the denominator consisted of administrative authorizations, they were not interpreted as patient-level case-fatality estimates.

Descriptive analyses included absolute and relative frequencies, cumulative totals, annual rates, and absolute and percentage changes between consecutive years. Means, medians, minima, maxima, standard deviations, and coefficients of variation summarized the indicators.

Temporal trend analyses were performed using the Joinpoint Regression Program, version 6.0.1 (U.S. National Cancer Institute, Bethesda, MD) [9]. Separate models were fitted for each combination of geographic region, valve-procedure category, and outcome, yielding 10 approved-AIH rate series and 10 in-hospital death rate series, each comprising 10 annual observations from 2015 to 2024.

Continuous piecewise log-linear models were used, with calendar year as the explanatory variable and the natural logarithm of the annual population-based rate as the response variable. All input rates were positive; therefore, no correction for zero values was required. The minimum number of joinpoints was set at zero and the maximum at two. At least two observations were required between each series endpoint and the nearest joinpoint, excluding the joinpoint itself, and at least two observations were required between consecutive joinpoints, excluding both joinpoints. Thus, when joinpoints were present, the model required at least three observations in each terminal segment and four observations in an internal segment when the joinpoint years were included.

The final model was selected using the weighted Bayesian information criterion. Annual percentage change (APC) was calculated for each segment as $\mathrm{100 {\times} [\exp(\beta) {-} 1]}$, where β represented the estimated slope on the natural logarithmic scale. Average annual percentage change (AAPC) summarized the 2015-2024 interval as $100 \times \left\{\exp\left[\frac{\sum_i w_i\beta_i}{\sum_i w_i}\right]-1\right\}$, where wᵢ represented the duration of each segment in years. Segment durations summed to nine elapsed years across the 10 annual observations. For models without joinpoints, the AAPC was numerically identical to the APC. Estimates were presented with 95% confidence intervals (CIs) calculated using the empirical quantile method. Statistical significance was assessed at a two-sided alpha level of 0.05.

The study used publicly available, aggregated data without individual identifiers. Under Brazilian National Health Council Resolution No. 510/2016, Article 1, sole paragraph, item V, research using aggregate databases without the possibility of individual identification is exempt from registration and review by the CEP/CONEP research ethics system [10].

## Results

Hospital admissions and in-hospital outcomes of native-valve IE

Between 2015 and 2024, Brazil recorded 13,920 AIH-based hospital admissions for native-valve IE treatment and 1,966 in-hospital deaths, corresponding to 14.1 deaths per 100 approved AIHs. Admissions were concentrated in the Southeast (n = 6,445; 46.3%) and Northeast (n = 3,924; 28.2%), followed by the South (n = 2,011; 14.4%), Central-West (n = 920; 6.6%), and North (n = 620; 4.5%). The Southeast accounted for most deaths (n = 1,070; 54.4%) and had the highest cumulative number of deaths per 100 AIHs (16.6), whereas the Northeast had the lowest (10.5; 412 deaths). Annual admissions peaked in 2016 (n = 1,727) and reached their minimum in 2020 (n = 1,126). Annual deaths ranged from 171 in 2016 to 247 in 2023. The Southeast had the highest mean admission rate (0.740 per 100,000 population), and the North had the lowest (0.320).

The mean hospital length of stay was highest in the Southeast (21.1 days), followed by the Central-West (19.0 days), North (18.7 days), South (18.0 days), and Northeast (17.8 days). The annual mean length of stay decreased between 2015 and 2018, followed by an increase in 2019 (+4.18%), a further decline in 2020 (-12.43%), and subsequent increases in the following years, including a 23.34% rise between 2021 and 2022. The highest value in the time series was recorded in 2024.

Joinpoint analysis identified a significant decline in admission rates in the Northeast during 2015-2019 (APC = -27.12%; 95% CI: -49.18 to -13.51), followed by a nonsignificant increase through 2024. In the Southeast, rates showed no significant change during 2015-2020, followed by a significant increase during 2020-2024 (APC = 8.24%; 95% CI: 4.73-16.14). We identified no joinpoints or statistically significant trends in the North, South, or Central-West (Figure 1).

**Figure 1:**
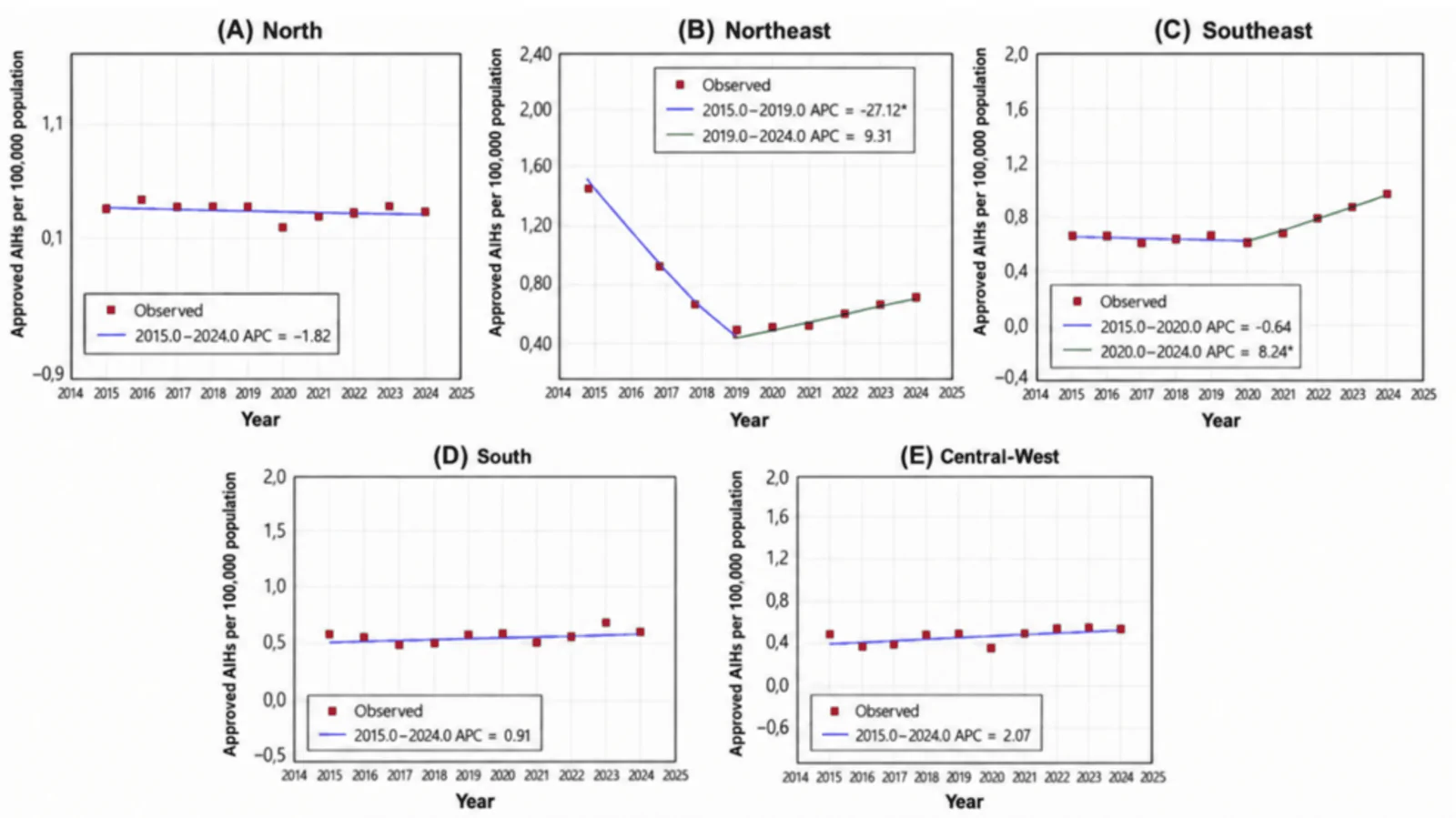
Annual approved AIH rates per 100,000 population for native-valve infective endocarditis treatment, Brazil, 2015-2024: (A) North; (B) Northeast; (C) Southeast; (D) South; and (E) Central-West. Red squares represent observed rates, and lines represent Joinpoint estimates. An asterisk indicates an APC significantly different from zero at α = 0.05 Authors’ analysis of SIH/SUS–DATASUS data using the Joinpoint Regression Program (version 6.0.1; U.S. National Cancer Institute, Bethesda, MD) AIH: Hospital Admission Authorization; APC: annual percentage change; SIH/SUS: Brazilian Unified Health System Hospital Information System

Among the 13,920 hospital admissions, 1,966 in-hospital deaths were recorded, corresponding to 14.1% of admissions. The Southeast accounted for the largest proportion of deaths (54.4%), followed by the Northeast (21.0%), South (14.9%), Central-West (5.7%), and North (4.1%). The proportions of hospital admissions resulting in in-hospital death varied across regions and years. Mean length of hospital stay was highest in the Southeast (21.1 days), followed by the Central-West (19.0 days), North (18.7 days), South (18.0 days), and Northeast (17.8 days).

For in-hospital death rates, no joinpoints were identified in any region. Significant increases occurred in the Southeast (APC = 3.27%; 95% CI: 0.94-5.56) and South (APC = 4.97%; 95% CI: 0.08 -10.09). No statistically significant trends were detected in the North, Northeast, or Central-West (Figure 2).

**Figure 2:**
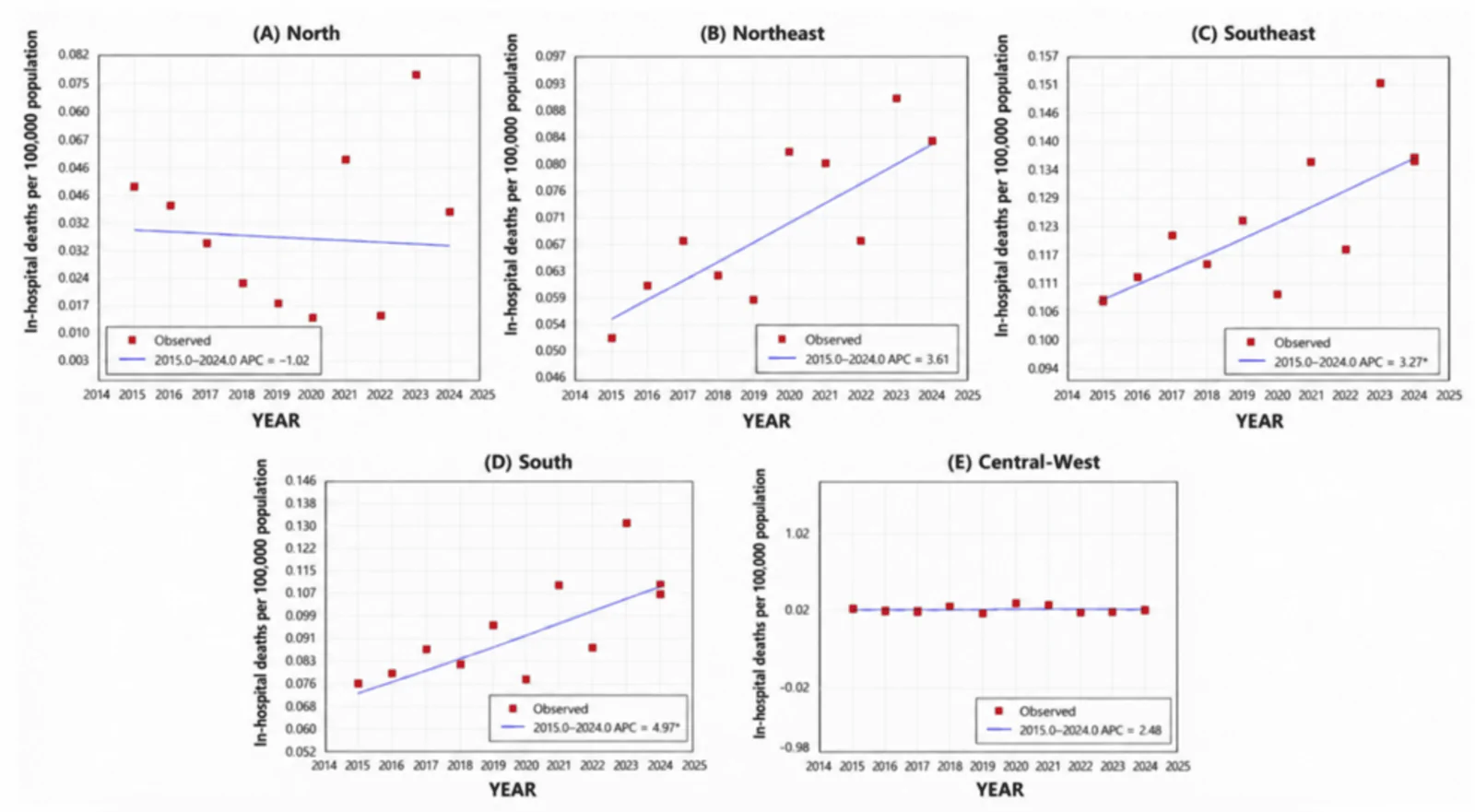
Annual in-hospital death rates per 100,000 population associated with native-valve infective endocarditis treatment, Brazil, 2015-2024: (A) North; (B) Northeast; (C) Southeast; (D) South; and (E) Central-West. Red squares represent observed rates, and blue lines represent Joinpoint estimates. An asterisk indicates an APC significantly different from zero at α = 0.05 Authors’ analysis of SIH/SUS-DATASUS data using the Joinpoint Regression Program (version 6.0.1; U.S. National Cancer Institute, Bethesda, MD) APC: annual percentage change; SIH/SUS: Brazilian Unified Health System Hospital Information System

Regarding variability in the indicators, the Northeast had the largest standard deviation in hospital admissions (SD = 192.90), followed by the Southeast (SD = 87.19), South (SD = 27.43), Central-West (SD = 13.94), and North (SD = 11.57). The highest coefficient of variation was observed in the Northeast (49.16%), whereas the lowest was observed in the Southeast (13.53%). For in-hospital deaths, the largest standard deviation was recorded in the Southeast (SD = 14.78), while the highest coefficients of variation were observed in the Central-West (47.21%) and North (44.88%).

Hospital admissions and in-hospital outcomes of prosthetic-valve IE

Between 2015 and 2024, Brazil recorded 6,610 AIH-based hospital admissions for prosthetic-valve IE treatment and 1,115 in-hospital deaths, corresponding to 16.9 deaths per 100 approved AIHs. Admissions were concentrated in the Southeast (n = 2,974; 45.0%), followed by the Northeast (n = 1,538; 23.3%), South (n = 1,222; 18.5%), Central-West (n = 493; 7.5%), and North (n = 383; 5.8%). The Southeast also accounted for the largest share of deaths (n = 530; 47.5%). Cumulative deaths per 100 AIHs ranged from 13.6 in the Central-West (67 deaths) to 17.8 in the Southeast.

Admission rates were generally highest in the South and lowest in the North, although the Southeast recorded the highest regional rate in 2024. Annual admissions and deaths were lowest in 2021 (499 and 86, respectively) and highest in 2024 (795 and 147, respectively).

Mean hospital length of stay was highest in the Southeast (20.7 days), followed by the Northeast (20.3 days), South (18.6 days), Central-West (18.4 days), and North (16.7 days). The highest annual value was recorded in the North in 2024 (24.2 days), whereas the lowest occurred in the same region in 2017 (11.0 days).

Joinpoint analysis identified three significant segments in the North: an increase during 2015-2017 (APC = 52.58%; 95% CI: 12.75-103.49), a decline during 2017-2021 (APC = -27.12%; 95% CI: -40.90 to -18.74), and a subsequent increase during 2021-2024 (APC = 24.80%; 95% CI: 2.91-75.56). In the Southeast, rates showed no significant change during 2015-2021, followed by a significant increase during 2021-2024 (APC = 20.04%; 95% CI: 3.90-47.44). No joinpoints or statistically significant trends were identified in the Northeast, South, or Central-West. Over the full study period, only the Southeast showed a significant overall increase (AAPC = 4.66%; 95% CI: 0.16-8.23) (Figure 3).

**Figure 3:**
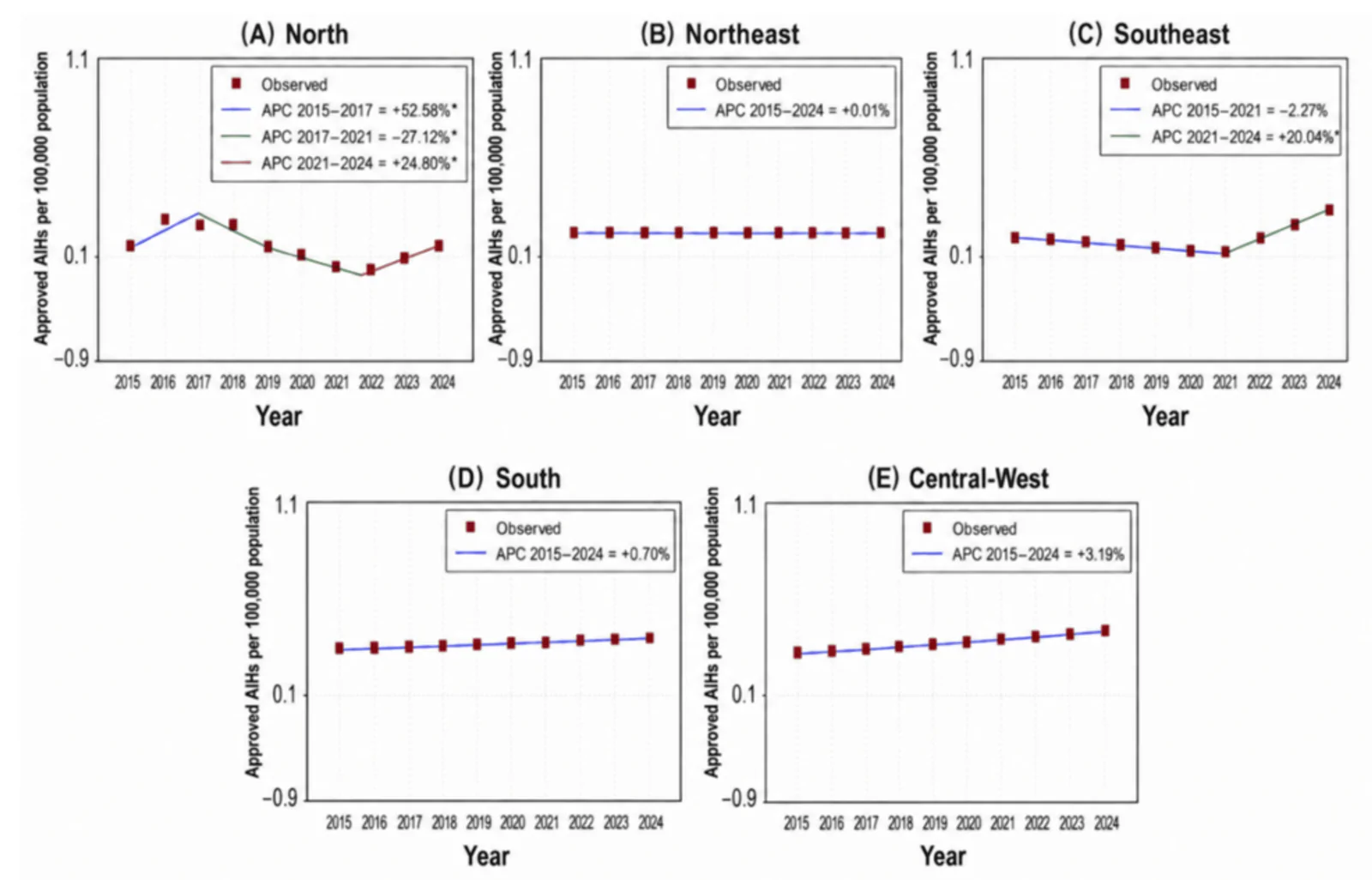
Annual approved AIH rates per 100,000 population for prosthetic-valve infective endocarditis treatment, Brazil, 2015-2024: (A) North; (B) Northeast; (C) Southeast; (D) South; and (E) Central-West. Red squares represent observed rates, and colored lines represent Joinpoint estimates. An asterisk indicates an APC significantly different from zero at α = 0.05 Authors’ analysis of SIH/SUS-DATASUS data using Joinpoint Regression Program v6.0.1 AIH: Hospital Admission Authorization; APC: annual percentage change; SIH/SUS: Brazilian Unified Health System Hospital Information System

During the study period, 1,115 in-hospital deaths were recorded, concentrated mainly in the Southeast (530), followed by the Northeast (248), South (207), Central-West (67), and North (63). The highest annual number of deaths occurred in 2024, with 147 records, whereas the lowest occurred in 2021, with 86 records. Annual changes in adjusted death counts showed substantial regional heterogeneity, particularly in the North, where deaths decreased by 72.50% between 2022 and 2023 and increased by 245.45% between 2023 and 2024. Other notable increases occurred in the Northeast between 2018 and 2019 (60.00%), the Southeast between 2021 and 2022 (35.56%) and between 2022 and 2023 (27.87%), the South between 2016 and 2017 (85.11%), and the Central-West between 2023 and 2024 (139.29%).

The in-hospital mortality proportion among admissions varied throughout the study period, ranging from 6.79% to 26.85% in the North, 11.40% to 19.94% in the Northeast, 14.79% to 20.23% in the Southeast, 11.31% to 23.21% in the South, and 8.95% to 24.28% in the Central-West. In 2024, the corresponding proportions were 17.67% in the North, 15.97% in the Northeast, 19.65% in the Southeast, 19.48% in the South, and 15.76% in the Central-West. No joinpoints or statistically significant trends in in-hospital death rates were identified in any region. All 95% CIs for APC included zero (Figure 4).

**Figure 4:**
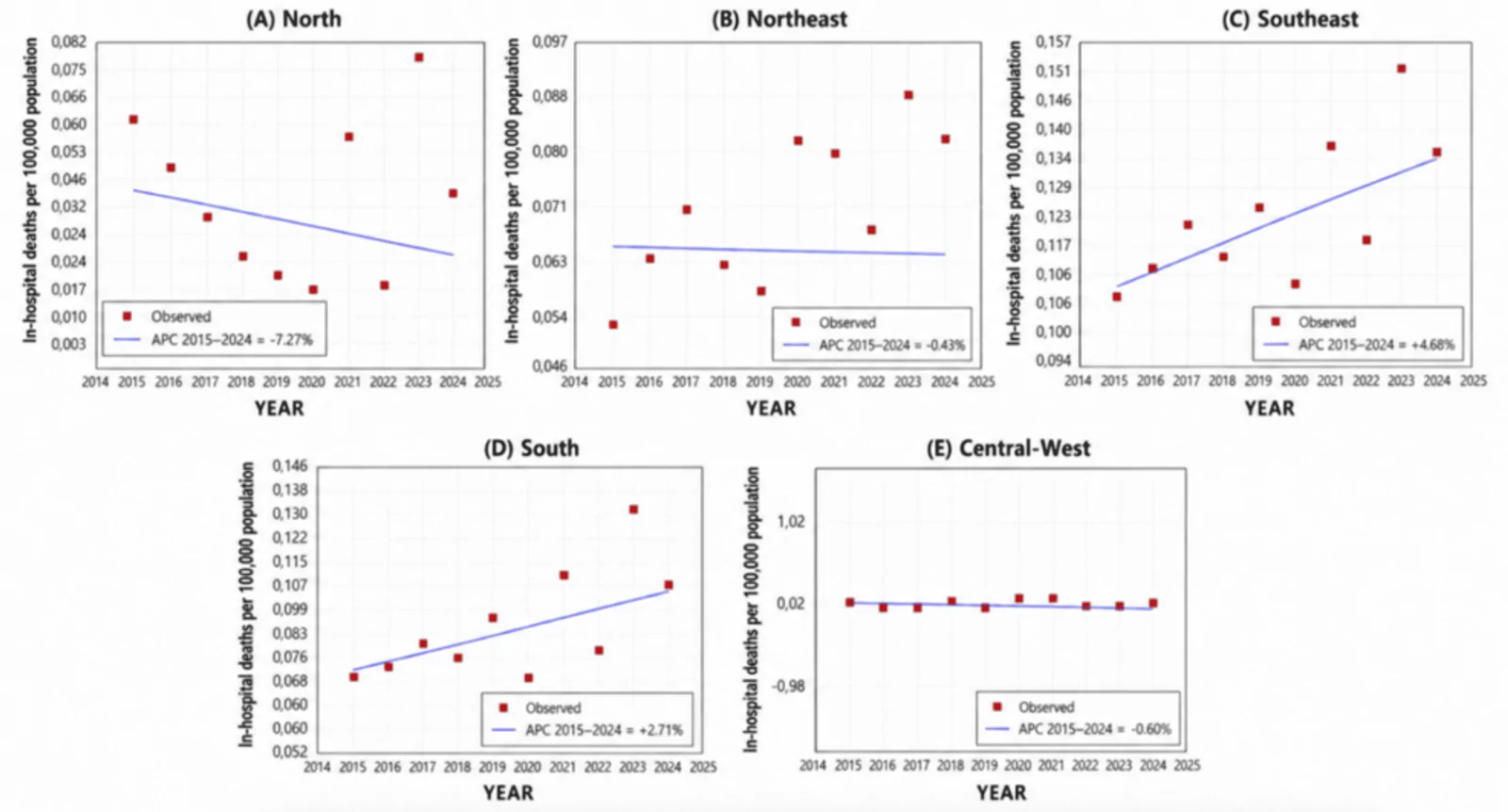
Annual in-hospital death rates per 100,000 population associated with prosthetic-valve infective endocarditis treatment, Brazil, 2015-2024: (A) North; (B) Northeast; (C) Southeast; (D) South; and (E) Central-West. Red squares represent observed rates, and blue lines represent Joinpoint estimates. None of the regional APC estimates was statistically significant at α = 0.05 Authors’ analysis of SIH/SUS–DATASUS data using the Joinpoint Regression Program (version 6.0.1; U.S. National Cancer Institute, Bethesda, MD) APC: annual percentage change; SIH/SUS: Brazilian Unified Health System Hospital Information System

Regarding variability in the indicators, the Southeast had the largest standard deviations for both hospital admissions (SD = 62.16) and in-hospital deaths (SD = 13.61), indicating greater absolute dispersion in the annual counts. The highest coefficient of variation for hospital admissions was observed in the North (CV = 40.64%), whereas the lowest was observed in the Northeast (CV = 11.44%). For in-hospital deaths, the Central-West showed the greatest relative variability (CV = 37.26%), followed by the North (CV = 32.66%), while the lowest coefficients were observed in the Northeast (CV = 22.07%) and South (CV = 23.89%).

## Discussion

This nationwide analysis identified regional heterogeneity in recorded hospital admissions and in-hospital deaths associated with IE treatment within SUS. NVE accounted for more approved AIHs, whereas PVE had a higher cumulative number of deaths per 100 AIHs. Recent increases in admission rates for both groups were concentrated in the Southeast. These findings extend Brazilian evidence derived largely from hospital series with uneven geographic representation [11].

The concentration of admissions in the Southeast and Northeast identifies where much of the recorded hospital care was delivered. Referral networks and differences in access to diagnostic and surgical services may contribute to this distribution. In EURO-ENDO, patients in lower middle-income countries presented later and received indicated cardiac surgery less frequently than those in high-income countries [12]. Although these international comparisons do not establish the mechanisms underlying regional differences in Brazil, they identify referral origin, diagnostic delay, and access to indicated surgery as important complements to regional admission rates when assessing service needs.

Temporal patterns also differed across regions. Recent increases in the Southeast contrasted with the earlier decline in NVE admission rates in the Northeast and fluctuations in PVE rates in the North. In a Brazilian referral-center cohort spanning four decades, Santos et al. documented increasing patient age and growing proportions of prosthetic-valve and healthcare-associated IE [13]. Global Burden of Disease estimates likewise describe increasing IE occurrence, particularly at older ages [14]. Changing age distributions and greater exposure to prosthetic materials are plausible contributors to hospital demand, although their contribution to the present regional trajectories was not measured.

National admission counts were lowest in 2020 for NVE and in 2021 for PVE, followed by recovery. This timing is compatible with disruption and subsequent resumption of care during the COVID-19 pandemic. Preveden et al. reported fewer monthly IE diagnoses during the pandemic in a Serbian center [15]. Nevertheless, the present data cannot distinguish changes in care-seeking, diagnostic activity, referrals, or hospital capacity. The decline in NVE admission rates in the Northeast also preceded 2020, indicating that pandemic-related disruption alone would be insufficient to explain the observed temporal variation.

Recorded NVE in-hospital deaths per 100,000 population increased in the Southeast and South. These trends should be assessed alongside admission volume and patient characteristics. EURO-ENDO identified associations between hospital mortality and heart failure, renal dysfunction, and intracardiac complications [16], while a Brazilian cohort demonstrated the prognostic value of age, heart failure, and creatinine at admission [17]. Conversely, the long-term Brazilian study reported declining adjusted hospital mortality despite an older population and a changing clinical profile [13]. Taken together, these findings show why increasing population-based rates of recorded hospital deaths cannot, by themselves, establish worsening individual prognosis.

PVE had a higher cumulative number of deaths per 100 AIHs than NVE, 16.9 vs. 14.1. Clinical studies offer possible explanations for this unadjusted difference. Pyo et al. reported more extensive perivalvular involvement, more abscesses, and higher early mortality among surgically treated patients with PVE [18]. The Spanish GAMES registry identified heart failure, persistent bacteremia, and intracardiac abscesses among factors associated with PVE mortality [19]. These associations provide clinical context but cannot be attributed to the present admissions without corresponding clinical data. The absence of statistically significant PVE death trends concerns change over time and therefore does not conflict with the higher cumulative ratio.

The Southeast had the longest mean hospital stays for both groups, approximately three weeks, emphasizing the relevance of IE to bed utilization and continuity of treatment. The POET trial demonstrated that partial oral antibiotic treatment was noninferior to continued intravenous treatment in selected, clinically stable patients with left-sided IE [20]. The 2023 European Society of Cardiology guidelines also support outpatient treatment for appropriately selected patients within structured follow-up pathways [21]. This evidence makes coordination between inpatient treatment and subsequent care a relevant consideration for SUS. Whether such pathways can safely reduce hospital utilization locally depends on clinical eligibility and the capacity to provide reliable follow-up.

This ecological analysis has limitations inherent to its administrative data source. Selection by treatment procedure codes may exclude admissions recorded under other codes, and the classification could not be clinically validated, introducing potential selection and misclassification bias [22]. Aggregated AIHs could not be linked to distinguish unique patients or hospitalization episodes from readmissions, transfers, and additional authorizations [7]. Regional comparisons may be influenced by hospital-location assignment, referral flows, population age structure, SUS coverage, and coding practices; population denominators do not eliminate these differences. Findings are restricted to SUS-funded care [7]. Individual diagnostic criteria, comorbidities, microbiology, echocardiographic findings, treatment details, and severity were unavailable for clinical validation or risk adjustment. Length of stay cannot establish clinical severity, and recorded deaths neither identify IE as the underlying cause nor capture postdischarge mortality. Consequently, these records do not provide estimates of population incidence, prevalence, or patient-level case fatality. Finally, 10 annual observations, small regional counts, and short Joinpoint segments limit precision; multiple comparisons warrant caution, and nonsignificant findings do not establish stability.

The study’s principal strengths are its national scope, 10-year observation period, inclusion of all five Brazilian regions, and separate evaluation of records linked to native-valve and prosthetic-valve treatment procedures. Applying the same procedure-code selection criteria across regions and years provided a common framework for examining recorded hospital activity. Reporting absolute counts alongside population-based rates, deaths per 100 approved AIHs, and mean hospital stay also captured complementary aspects of utilization and recorded outcomes. These features extend the geographic perspective available from individual hospital series and provide a reference for subsequent monitoring of the selected treatment procedures within SUS.

## Conclusions

Hospital records linked to the selected SIH/SUS procedure codes for native-valve and prosthetic-valve IE treatment showed distinct regional and temporal patterns during 2015-2024. The native-valve treatment category accounted for more approved AIHs and recorded in-hospital deaths, whereas the prosthetic-valve treatment category had a higher cumulative number of deaths per 100 AIHs. Authorizations were concentrated in the Southeast and Northeast, and the Southeast had the longest mean hospital stays for both treatment categories. Recent increases in approved AIH rates in the Southeast contrasted with the earlier decline in the Northeast for the native-valve treatment procedure. Rates of recorded in-hospital deaths per 100,000 population increased in the Southeast and South for the native-valve treatment category, while no statistically significant regional trends were identified for the prosthetic-valve treatment category.

The geographic distribution of authorizations, cumulative deaths per 100 AIHs, and temporal changes provide complementary information about hospital activity recorded under these procedure codes. The higher cumulative death-to-AIH ratio for prosthetic-valve treatment coexisted with an absence of statistically significant death-rate trends, supporting separate monitoring of these indicators. Although these administrative records cannot establish disease incidence or clinical severity, they provide a basis for monitoring hospital utilization associated with the selected treatment procedures and informing capacity planning within SUS using AIH volume, recorded deaths, and length of stay.
